# A Multi-Center, Randomized, Blind, Controlled Clinical Trial of the Safety and Efficacy of Micro Radio Frequency Therapy System for the Treatment of Overactive Bladder

**DOI:** 10.3389/fmed.2022.746064

**Published:** 2022-05-12

**Authors:** Zhi-Hui Xu, Peng-Fei Zhang, Yu-Feng Wang, Ao Ma, Yasmeen Bano, Alisherjon Ibrohimov, Chen Zhang, Hao-Fei Jiang, Yang Zhang, Yan-Lan Yu, Hai-Hong Jiang

**Affiliations:** ^1^Zhejiang Provincial People's Hospital, Hangzhou, China; ^2^The First Affiliated Hospital of Wenzhou Medical University, Wenzhou, China; ^3^Zhejiang-California International NanoSystems Institute, Hangzhou, China; ^4^Sir Run Run Shaw Hospital, School of Medicine, Zhejiang University, Hangzhou, China

**Keywords:** micro radiofrequency, overactive bladder, urinary incontinence, urgency, minimally invasive

## Abstract

**Purpose:**

The purpose of this study was to evaluate the efficacy and safety of low power micro radiofrequency (RF) therapy (μRFthera^®^) through urethra in the treatment of overactive bladders (OAB) through a prospective, single-blind, placebo-controlled, multi-center clinical protocol.

**Materials and Methods:**

One hundred and fourteen patients with refractory OAB were randomized at 2:1 ratio, treatment to control undergoing same procedures except only the micro-RF treatment group at turned “on” setting in energy. Bladder diaries recorded during the screening period (3 days before enrollment) and during follow-up period on week 1, 3, and 7, respectively. The patients in control could choose receiving an energized treatment during extension stage.

**Results:**

The treatment efficacy was 76.1%. There was 49.80% rate improvement compared to control (95%CL 32.48%, 67.13%). The crude rate ration (RR) was 2.89, 95% CI (1.67–5.01) with *p* < 0.001 in uni-variate analysis, while the RR became 2.94, 95% CI (1.67–5.16) with *p* < 0.001 after adjusted potential confounding factors in multi-variate analysis. Statistically significant improvements have been demonstrated in the frequency of urination, urgency, nocturia, and quality of life (QoL) scores.

**Conclusions:**

Micro RF therapy is safe and effective for the treatment of OAB. The main treatment-related complications were catheterization related complications.

**Clinical Trial Registration:**

Zhejiang Device Registration Certificate No. 202090909, www.chictr.org.cn, Clinical Trial Accession Number: ChiCTR2100050096.

## Introduction

Overactive bladder (OAB) is a chronic medical condition characterized by symptoms of urinary frequency and urgency, with or without symptom of urgency urinary incontinence (1). OAB prevalence is widespead. It was estimated more than 500 million people suffering from OAB (2). A randomized community-based, cross-sectional study showed that the overall prevalence of OAB in China was 2.1% (3). A population-based, cross-sectional survey showed that the overall prevalence of OAB was 11.8% in Canada and four other European countries (4). While a cross-sectional, population-representative survey showed that the prevalence of OAB in America was 25% in men and 50% in women and exacerbates with aging (5). The medical costs of patients with OAB are more than 2.5 times higher than those of similar patients without OAB, placing a serious burden on both the patient families and social welfare (6). In addition to patient quality of life (QoL), OAB affects workforce productivity and is also associated with an increased tendency to anxiety and depression (7, 8).

The first-line OAB treatment options includes behavioral therapies, followed by pharmacotherapy as second-line treatment. More intrusive solutions include intravesical drug instillation, multi-point bladder injection of botulinum toxin type A, sacral neuromodulation, and other surgical treatments (9). However, behavioral treatments, such as bladder training and Kegel exercises, have long treatment cycle and poor patient compliance. The efficacy of pharmacotherapy is limited with low tolerance in practice (10). Surgery and neuro-modulation therapy are invasive approaches that may have to be performed in stages and have surgical realated problems (11, 12). These limitations cause many middle-aged and elderly OAB refractory patients lack of effective treatment and seriously affect the QoL. Therefore, there is urgent need innovative treatment options, especially non-invasive or minimal invasive, effective, safe, and economical theapies. Radiofrequency (RF) therapy and treatment have these characteristics with many fields of application in surgery and medicine (13, 14). It has been applied to a variety of diseases as an economical, efficient and minimally invasive treatment option with high compliance (15).

A micro-RF trerapy related to this study is a trans-urethral procedure using multi-polar μRFthera technology with designed delivering a max power of 2.5 W to the bladder wall. The temperature is controlled under 45°C which may reduce the sensitivity of the submucosal nerve endings of the bladder (16). Therefore, we applied for this clinical trial to evaluate the efficacy and safety of RF therapy system in the treatment of OAB.

## Method and Analysis

### Study Design

**Figure 1:**
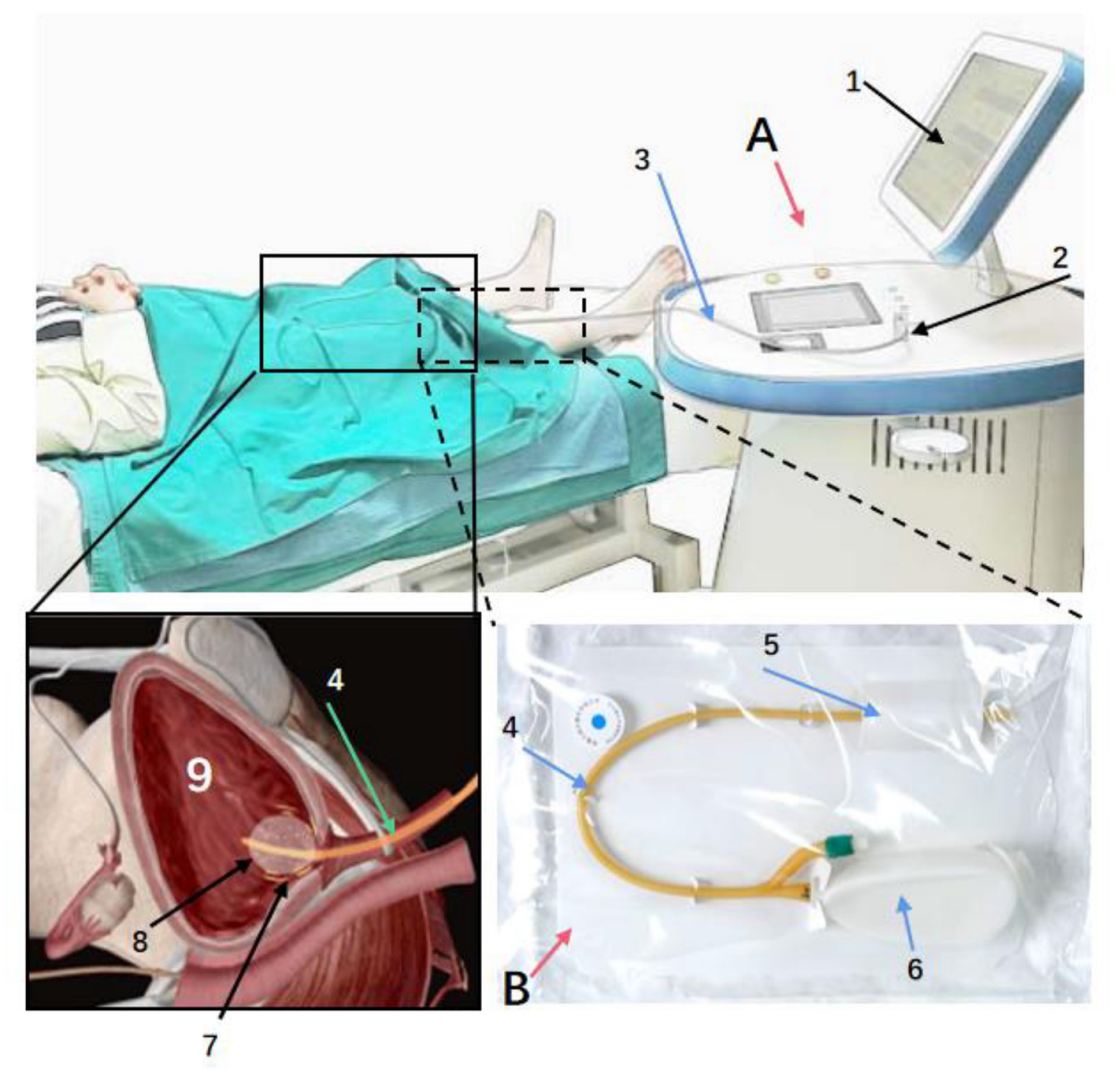
The product is mainly composed of two parts: therapeutic apparatus **(A)** and catheter **(B)**. Through therapeutic electrodes integrated with the surface of the urinary catheter, the micro radio frequency energy is delivered to the nerve fibers under the bladder wall, blunting part of the nerve endings temporarily. **(A)**: a: Display. b: Power Switch. c: Barcode Scanner. d: RF Output Connector. e: Rotary button to adjust treatment parameters. **(B)**: 1: Catheter. 2: Port integrated with Therapeutic Electrodes. 3. Port of RF Input.

This was a multicenter clinical trial aim to evaluate the safety and efficacy of μRFthera^®^ system (Manufactured by Rebeccatech Medical and Science Ltd). It was a hospital-based, prospective, multicenter, single-blind, randomized controlled clinical trial carried out in three tertiary hospitals. Details of the participating hospitals were listed in the Supplementary Table 1. The protocol and informed consent form were approved by the IRB of each site, and all study participants gave written informed consent prior to study participation. The product was validated by the Zhejiang Medical Device Testing Institute and China Food and Drug Administration (Report No. Z20183035-D, Z20182736) prior to the study. The structural composition of the product has seen in Figure 1, where the therapeutic apparatu and the single use modified catheter is displayed.

Following the protocol, 114 OAB patients were to be recruited from the three centers (experimental group to control group, 76: 38). For a specific center, the final enrolled patients should not be >25% or more than 50% of the total number of subjects. In stage 0 (screening phase), each subject was randomly assigned to the experimental group or the control. Baseline assessments were performed 3 days before the beginning of the therapy and data checked by assigned investigators (urologists). In stage 1 (core trial phase), the subjects in both experimental group and control group were treated with the RF therapy system. The only variance was that the micro-energy was “on” for the experimental group, and “off” for the control. Each subject received two times treatments separated by seven days, each with 20 min. Treatment was administered after enrollment (Week 0) and at Week 1. Follow-up was at one, three, and seven weeks, and the main evaluation endpoint was at the end of the week 7 (or 6 weeks after the 2nd treatment).

During the treatment procedure, the patient was in a supine position, the investigator (urologist) completed catheterization. The RF cable connected the catheter via the connector to the RF output connector of the therapeutic apparatus (Figure 1). The investigator started the instrument for 30 min treatment along with observing and asking the patient whether there was any discomfort. After the treatment, the investigator turned off the machine, disconnected the electrodes, and removed the modified catheter immediately.

**Figure 2:**
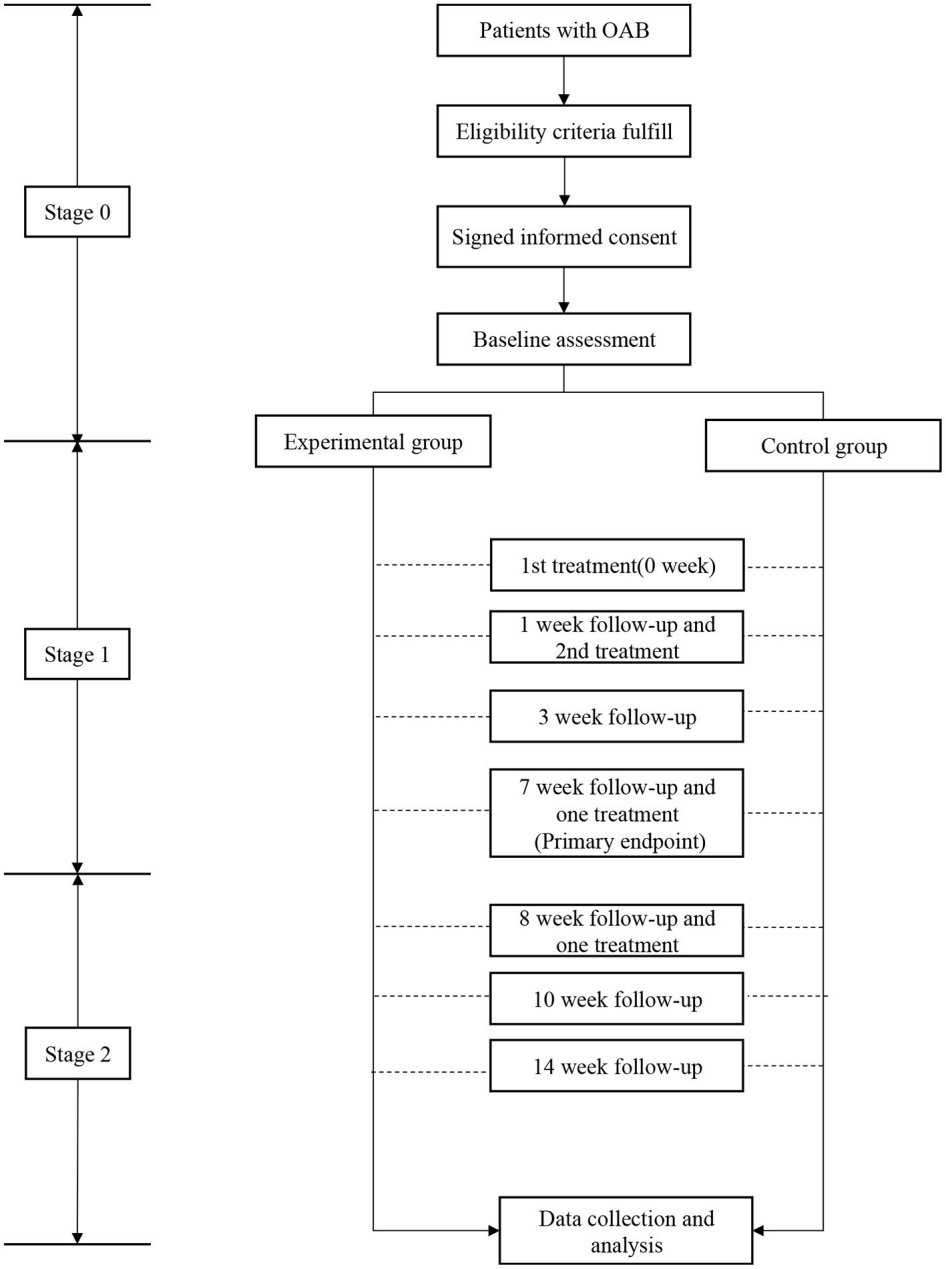
The study flow chart. During stage 0, after checked for eligibility, subjects were required to sign the informed consent followed by baseline assessment. During stage 1, subjects were randomly divided into control or treatment groups with 14 weeks followup.

In stage 2 (the extension phase), patients in control may choose to receive the energized treatment with micro-energy “on” in the treatment. The IRB committee deemed the treatment in extension phase have potential benefits to the patients. In stage 2, the patients were treated at 7th and 8th week, and followed up at 8th, 10th, and 14th week. The detailed study flow chart was shown in Figure 2.

### Biases/Blinding

The randomization sequence (2:1 ratio) was developed using the PROC PLAN process of SAS 9.4 with six patients as minimum cohort. Allocation concealment was conducted using sealed opaque envelopes, distributed by the Clinical Research Associate upon the enrollment of each subject, to ensure that each subject has an equal opportunity of allocation. Because the evaluation of the treatment effect of OAB was based primarily on the bladder diary completed by the subject and was vulnerable to subjective factors, patients were blinded to the treatment approach (17). Investigators did not participate in screening, device manipulation, but participate in performing the “bladder diary” assessment with in blinding way.

### Inclusion and Exclusion Criteria

Patients at least 18 years old with OAB were recruited for further assessment of clinical history and basic information, including primary complaints, OAB history, surgical history, chronic coexisting diseases, and current medication. Urinary ultrasound were examined to rule out patients with secondary OAB, and urinalysis performed to rule out hematuria and acute urinary tract infection. The Supplementary Table 2 showed a detailed list of the main inclusion and exclusion criteria. During the process of the trial, subjects who withdrew from the study for any reason should recorded the Case Report Form (CRF, the Supplementary Table 3). The Supplementary Table 4 showed a detailed list of the main discontinuation criteria.

### Primary Outcomes

The primary outcome was the number of successful treatment cases. The success rates of the experiment group and the control were calculated separately, and the differences between the two groups were compared.


$$\begin{array}{lll} \text{Success rate} & = & \frac{\text{the number of successful cases }}{\text{total number of cases}}\times 100\% \end{array}$$

Patients completed bladder diary 3 days before each visit or treatment at Week 0, Week 1, Week 3, Week 7. The primary outcome was comparing the improvement from baseline at the 7-week follow-up. Success is defined as meeting any of the following criteria, including:

A. ≥50% decrease or normalization (<8 times/day) from baseline in the average number of 24-h micturitions;

B. ≥50% decrease or normalization (none) from baseline in the average number of 24-h urge incontinence;

C. ≥50% decrease or normalization (none) from baseline in the average number of 24-h urgency.

### Secondary Outcomes

Secondary outcomes were the changes from baseline in mean volume per micturition per 24 h and quality of life (QoL) (18). Patients completed bladder diary and QoL score charts 3 days before each visit or treatment at Week 0, Week 1, Week 3, Week 7, Week 8, Week 10, Week 14. Among them, Week 8, Week 10 and Week 14 were only applicable to patients who completed stage 2.

### Sample Size Calculation

The trial was a prospective, multicenter, single-blind, placebo-controlled superiority trial design. There was no directly available clinical data of similar products that can be used to judge the clinical success rate of this product. At present, sacral neuromodulation, as a treatment for refractory OAB, was recognized in the field as its therapeutic effect was about 40% (19). Sacral neuromodulation may inhibit the afferent activity of the bladder by stimulating the somatic afferent component of the sacral nerve, which have potential a closest therapeutic mechanism we could obtain as comparison.

Therefore, we determined the expected treatment success rate in the trial at 40%, the bound value of efficiency test was 0, and the treatment success rate was conservatively estimated to be 70% according to the preliminary testing results. When significance level was 0.025 on one side, power of the test was 0.8, and the allocation ratio among groups was 2:1, which was calculated by $n_{c}=\frac{{\left(Z_{1-\alpha }+z_{1-\beta }\right)}^{2}}{{\left(\delta -\Delta \right)}^{2}}\left[\frac{T(1-T)}{K}+C\left(1-C\right)\right]$. It was calculated 60 valid cases to be completed in the experimental group and 30 valid cases in control. Considering about 20% dropout rate, a total of 114 subjects were pre-enrolled.

K: Grouping proportion; n_c_: Sample size of control group; n_t_= K n_c_ Sample size of experimental group;

T/C: Expected treatment success rate of experimental group and control group; δ = T-C; Δ = 0.

### Statistical Methods

All statistical analyses were conducted with SASV. 9.4 and R package. Statistical significance is defined as *p* < 0.05. Basic demographic characteristics, vital signs, etc. were analyzed to illustrate whether the basic situations of the two groups were comparable. Quantitative indicators, such as number of cases, mean, standard deviation, median, maximum and minimum, the *t*-test or Wilcoxon rank-sum test, were calculated. Qualitative indicators, such as treatment success rate, were analyzed by χ2 test or Fisher's test. The difference in the overall success rate between the two groups were compared using the χ2 test or Fish exact probabilities, as well as the 95% confidence interval of the difference in success rate between the two groups.

### Quality Control

Site investigators and research staff must complete a training program on study design and processes to achieve standardization and data recording consistency. All participants agreed to ensure the accuracy of the data and the compliance of the study protocol. For quality control of serum and urine sample testing, each site's clinical laboratory must meet ISO15189 and/or CAP requirements. An independent data and safety monitoring committee monitored the precision and scientific integrity of the trial. Interim meetings were held every 2–3 months to ensure adherence to the protocol and proper data collection.

### Patients and Public Involvement Statement

Research protocols and outcome measures were developed by referring to the symptoms and characteristics of patients with OAB. Although patients did not participate in the design phase of the study, their concerns were addressed during recruitment and study conduct.

## Results

### Study Participants

This study was completed in all three centers by June 2020. A total of 114 cases were enrolled with informed consent. The experimental group and the control group were enrolled 2:1 (76:38). All subjects were FAS effective analysis, a total of 9 cases were removed from the PPS analysis, the results of the total analysis set of 114 cases, including 76 cases in the trial, and 38 cases in the control; 105 cases in the protocol set, with 67 cases in the trial, and 38 cases in the control; All cases were included in the SAS safety analysis set.

Baseline characteristics were balanced between groups. Overall 85.1% of patients were women. No significant difference was observed in the two groups in terms of sex, ethnicity, age, height, weight, temperature, heart rate and blood pressure. The comparison of the general information of the two groups was shown in Supplementary Table 5.

### Compliance and Device Use Analysis

Patient compliance were 89.47% in the experimental group and 100.00% in control. The difference between the two groups was not statistically significant. Equipment exposure in the experimental group and the control group were the same, and there was no statistical significance in the use parameters and the convenience of operation during the use.

### Primary Efficacy

Primary efficacy was the inter-group comparison of the success rate of successful treatment (Supplementary Table 6). The FAS analysis results after treatment demonstrated the success rate of 76 patients in the experimental group was 67.1%, compared with the 26.3% of 38 patients in the control group. The success rate difference between the two groups was estimated to be 40.79% (95%CL 23.35%, 58.33%; *P* < 0.01); After eliminating the cases meeting the exclusion criteria, PPS analysis results demonstrated the success rate of the experimental group was 76.1%, compared with the 26.3% of the control group, the success rate difference between the two groups was estimated to be 49.80% (95% CL 32.48%, 67.13%; *P* < 0.01); Since five cases were excluded due to missing visit follow-up at week 7, the difference of success rate between the two groups was estimated to be 34.21% (95%CL 16.41%, 52.01%; *P* < 0.01); According to the analysis of PPS, the crude success rate ratio (RR) was 2.89, 95%CI (1.67–5.01) with *P* < 0.001 in univariate analysis. The success RR become 2.94, 95%CI (1.67–5.16) with *P* < 0.001 after adjusted for potential confounding factor: sex, age, pre-treatment frequency urination in multivariate analysis. The lower limits of the rate differences above are much higher than 0, indicating that the experimental group has significantly better therapeutic effect than the control group.

After the corrections of center effect, baseline frequency of urination, duration of OAB, and combined treatments, the logistic regression model was used to test the effect between treatment groups (*P* < 0.01). It indicated the experimental group was more effective than the control. According to the results of FAS analysis, PPS analysis and sensitive set analysis were consistent.

Breslow-day test of the above three analysis sets *p* > 0.05. This indicated the consistency between centers; In the Logistic model, the interaction terms between centers and groups indicated no statistical significance, all demonstrating that the results among the three centers are consistent.

### Secondary Efficacy

Prior to treatment frequency of urination, urinary incontinence, urgency and nocturia were similar between the two groups, and the differences were not statistically significant. Post-treatment urinary performance data were all statistically significant between groups. In the experimental group, the frequency of urination decreases by 4.16 times per day and the frequency of urgent urination decreases by 5.41 times per day, both of which indicated significant improvement (*P* < 0.01); Nocturia frequency decreases significantly in the experimental group, with a mean decrease of 1.16 times per day (*P* < 0.05); The quality of life scores in the experimental group improve significantly, with an average decrease of 2.11 points (*P* < 0.01) (Supplementary Table 7). There was no significant difference in the change of urinary residual volume between the groups.

### Safety Analysis

A total of 47 cases (41 cases) of adverse events were recorded in the study, with a total incidence of 36.0% (41/114). The incidence of adverse events in the experimental group was 28.9% (22/76), and that in the control was 50.0% (19/38). The incidence of adverse events related to micro-RF therapy devices was 11.8% (9/76) in the experimental group and 26.3% (10/38) in the control. Only one case of serious adverse reactions (SAE), unrelated to the device, occurred in the control, and no other SAEs were observed.

Discontinuations or withdrawals from the study were rarely due to use of the product. There were 19 cases of product-related adverse events, with an incidence of 16.7%. Adverse events between the groups were mainly injuries to the urinary tract during catheterization, resulting in urinary tract infection or bleeding. Most of the symptoms were relieved or disappeared after day 3. Post treatment, there was no significant difference in the proportion of normal to abnormal laboratory indicators between the two groups compared to baseline. Urinalysis abnormalities caused by urinary tract infection were common in both groups, and there was no statistically significant difference between the two groups. The incidence of adverse events in both groups were similar (Supplementary Table 8).

## Discussion

This study is the first prospective, single-blind, randomized controlled clinical trial to verify the efficacy and safety of micro-RF therapy devices for OAB. After treatment, patients in the experimental group had statistically significant clinical effects in reducing OAB symptoms compared to the control. In addition, no unacceptable complications were observed throughout the study.

Currently, behavioral therapy and medication remain the preferred first-line treatment options for patients with OAB. However, both of these therapeutic methods have obvious clinical limitations, especially for patients with poor adherence, pregnancy, and drug intolerance. Traditional, high-temperature RF therapy system have been applied to a variety of diseases as an economical, efficient and minimally invasive treatment method with high compliance. RF ablation of atrial fibrillation has been used for many years and has now become a common surgical approach (20). RF treatment of chronic vertebrogenic low back pain, primary trigeminal neuralgia and other related research are emerging (21–23). Relevant animal studies have shown that RF treatment reduces bladder nerve density without evidence of lasting epithelial injury (24). RF electrical stimulation of pelvic nerves has a long-lasting inhibitory effect on the 0.5% acetic acid-induced detrusor overactivity rat model without causing significant nerve injury (25). There is one study that reported long-term improvement in pain and symptoms associated with interstitial cystitis in patients after RF treatment of the superior hypogastric plexus (26). However, to the authors best knowledge from literatures, only one smaller trail (of 63 patients) conducted by Tu et al. (27) has evaluated the efficacy and safety of RF therapy for refractory OAB.

**Figure 3:**
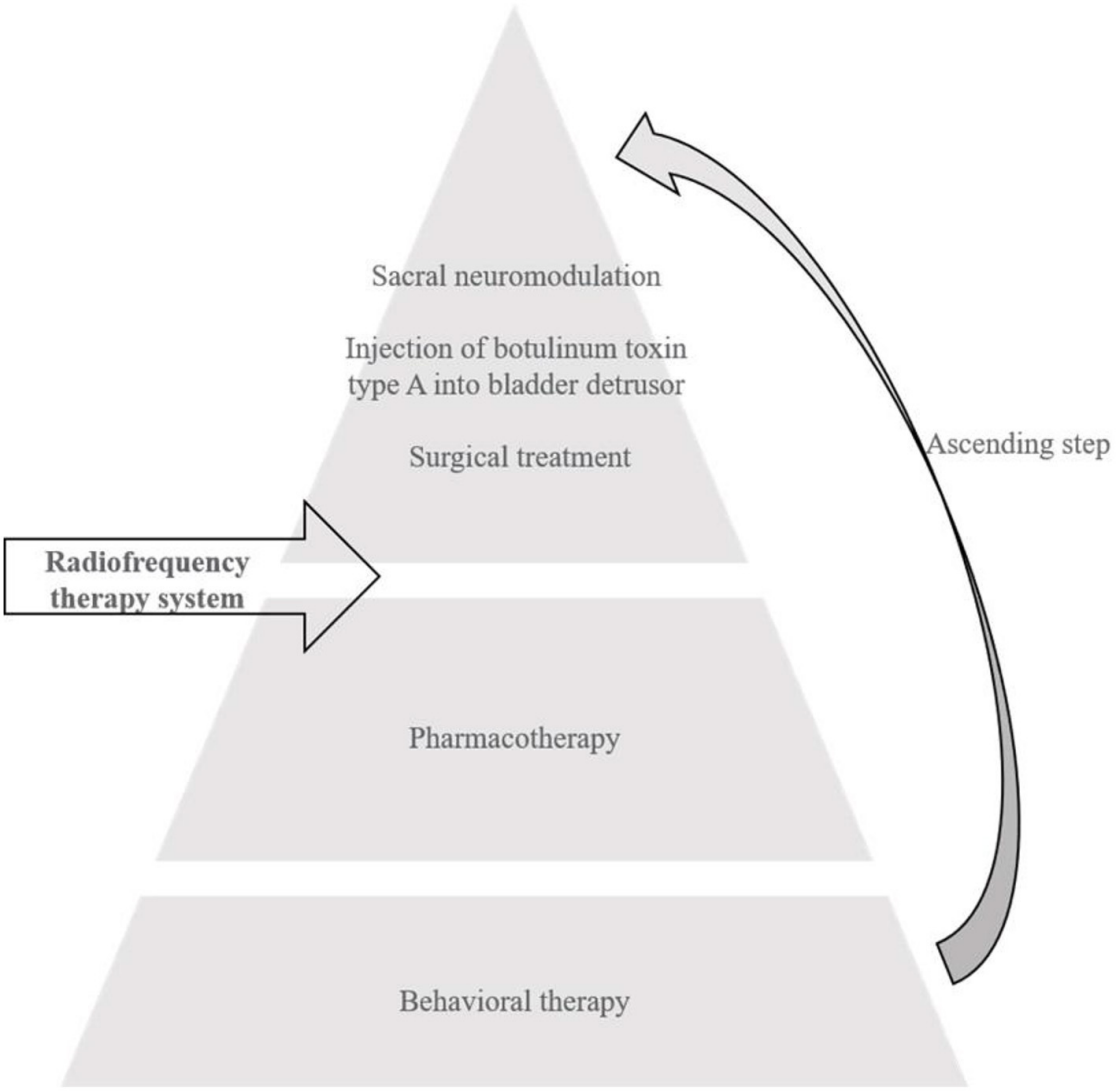
The projected sequence of RF therapy system in the treatments of OAB.

The micro-RF therapy we conducted in this study is purposefully different from any traditional high-temperature RF treatments. In most of the previous studies, RF energy at tens or hundreds of electric watts were injected into organs and tissues, resulting in working temperature much higher than 60°C. The micro-RF therapy controls the RF power under 10 W and maintains the treatment temperature at below 45°C with a precision energy delivery antenna system. We conducted this controlled study to validate this minimally invasive approach be more advantageous to traditional RF therapy. Therefore, we think the sequence of micro-RF therapy system in the treatments of OAB in future as its efficacy and safety with the characteristics of the non-/mini-invasive approach (Figure 3).

In the trial, patients receiving RF therapy had an success rate of 67.1% at week 7, significantly higher than the expected treatment success rate of 40%. In comparison, the anticholinergic tolterodine response rate was 54.8% in a 12-week randomized controlled trial (28). In terms of secondary indicators, the frequency of voiding, urgency, nocturia, and quality of life score were statistically significant between the groups after treatment. The safety of RF therapy was also reliable from the study results. The incidence of adverse events related to RF therapy devices in the experimental group was 11.8%, which was far lower than the 57.9% of anticholinergic therapy (29), 38.1% of onabotulinumtoxinA by injection (30), and 30% of sacral neuromodulation (31). The micro-RF treatment of OAB demonstrated a relative high response rate with a low and tolerable adverse event rate, although its long-term efficacy and complication still remains to be verified.

From the study as the trial evaluating therapeutic devices, we designed a single-blind randomized controlled trial to reduce possible bias. At the same time, investigators who did not participate in screening or device manipulation but participated in the “bladder diary” assessment which was also blinded. In addition, including documenting the efficacy of micro-RF therapy systems; other evaluations, like QoL of patients, has also been verified. Admittedly, the study was also some limitations. It was a hospital-based multi-center recruition of patients with only from China which may still not be fully representative of the Chinese OAB patient population. Moreover, our recruitment being hospital-based may be biased toward those who suffer from moderated to severe OAB. Further potential disadvantages were that blood and urine samples were measured in each authorized hospital rather than in one central laboratory. The possibility of bias and variation between laboratories or examiners may exist. In addition, the results of this study were limited to 14 weeks of follow-up, and a long-term clinical trial for further study may required to confirm the efficacy and safety consistently.

## Data Availability Statement

The original contributions presented in the study are included in the article/Supplementary Material, further inquiries can be directed to the corresponding author/s and their GCP offices.

## Ethics Statement

The clinical trial protocol was reviewed and approved by the Institutional Ethics Committees of The First Affiliated Hospital of Wenzhou Medical University; Zhejiang Provincial People's Hospital; and Sir Run Run Shaw Hospital, School of Medicine, Zhejiang University. The patients/participants provided their written informed consent to participate in this study. Written informed consent was obtained from the individual(s) for the publication of any potentially identifiable images or data included in this article.

## Author Contributions

Z-HX, Y-LY, and H-HJ as principal investigators (PIs) were responsible for the design, project development, and management of the clinical trial. YZ was responsible for the debugging of the instrument. AM, Y-FW, P-FZ, and YB was responsible for data analysis and manuscript drafting. P-FZ, H-FJ, AI, CZ, and H-HJ was responsible for the manuscript editing, revising, submitting, and responding to comments. All authors read and approved the final manuscript.

## Funding

This study was supported in part by Zhejiang Provincial Natural Science Foundation of China (No. LWY20H050001 to H-HJ) and Zhejiang Provincial medical and health technology program projects of China (2019KY101 to H-HJ).

## Conflict of Interest

The authors declare that the research was conducted in the absence of any commercial or financial relationships that could be construed as a potential conflict of interest.

## Publisher's Note

All claims expressed in this article are solely those of the authors and do not necessarily represent those of their affiliated organizations, or those of the publisher, the editors and the reviewers. Any product that may be evaluated in this article, or claim that may be made by its manufacturer, is not guaranteed or endorsed by the publisher.

## Abbreviations

RF: radiofrequency
OAB: overactive bladders
QoL: quality of life
SAE: serious adverse event
CRF: case report form.
