# Supplementary material for: A Multi-Center, Randomized, Blind, Controlled Clinical Trial of the Safety and Efficacy of Micro Radio Frequency Therapy System for the Treatment of Overactive Bladder
Source: Front Med (Lausanne). 2022 May 12;9:746064. doi: 10.3389/fmed.2022.746064 (PMC9133845; doi:10.3389/fmed.2022.746064)
Supplement: Supplementary file 1 [file Table_1.pdf]

Supplementary Table 1: Study locations and participating hospitals

Partners	City/Province
Zhejiang Provincial People's Hospital (Leading site)	Hangzhou/Zhejiang
The First Affiliated Hospital of Wenzhou Medical University	Wenzhou/Zhejiang
Sir Run Run Shaw Hospital, Zhejiang University School of Medicine	Hangzhou/Zhejiang
