# Supplementary material for: A Multi-Center, Randomized, Blind, Controlled Clinical Trial of the Safety and Efficacy of Micro Radio Frequency Therapy System for the Treatment of Overactive Bladder
Source: Front Med (Lausanne). 2022 May 12;9:746064. doi: 10.3389/fmed.2022.746064 (PMC9133845; doi:10.3389/fmed.2022.746064)
Supplement: Supplementary file 2 [file Table_2.pdf]

Supplementary Table 2: the inclusion and exclusion criteria

Inclusion criteria (If the answer to any of the following is "No", the subject cannot participate in the clinical trial)
Age ≥ 18 years.
Patients meeting criteria for refractory OAB.
Normal upper urinary tract function, bladder capacity > 100ml.
Patients in persistent OAB who have or not been on medication or other therapies
Voluntary participation in the trial with written informed consent.
Able to communicate with the investigators and willing to comply with the requirements of the trial.
Exclusion criteria (If the answer to any of the following is "Yes", the subject cannot participate in the clinical trial)
Pregnant or lactating women.
Patients with secondary OAB symptoms such as urinary tract obstruction.
Patients with uncontrolled urinary system infection within 1 week.
Patients with renal insufficiency and serum creatinine greater than 1.5 times of normal value.
Patients with surgical contraindications.
Patients with any implanted neurostimulator, cardiac pacemaker or implantable defibrillator.
Presence of concomitant diseases seriously affecting health, such as malignant tumor.
Patients who have received botulinum toxin treatment in the past 12 months.
Patients allergic to latex materials.
Presence of other concomitant disease affecting the efficacy of the trial.
Patients who participated in other clinical trials 3 months before the trial.
Other conditions that the investigator considers inappropriate for the study.
