# Supplementary material for: A Multi-Center, Randomized, Blind, Controlled Clinical Trial of the Safety and Efficacy of Micro Radio Frequency Therapy System for the Treatment of Overactive Bladder
Source: Front Med (Lausanne). 2022 May 12;9:746064. doi: 10.3389/fmed.2022.746064 (PMC9133845; doi:10.3389/fmed.2022.746064)
Supplement: Supplementary file 3 [file Table_3.pdf]

Supplementary Table 3: Case Report Form (CRF)

Items	Stage 0	Stage 1			Stage 2			
		1st Treatment	2nd Treatment	Follow-up	3rd Treatment	4th Treatment	Follow-up	
		0 week	1 week	3 weeks	7 weeks	8 weeks	10 weeks	14 weeks
Signed informed consent	X							
Bladder diary	X		X	X	X	X	X	X
Inclusion and exclusion criteria	X							
Discontinuation criteria		X	X	X	X	X	X	X
Basic information[1]	X							
Vital Signs[2]	X							
Medical history	X							
Allergy history	X							
Family history	X							
Surgical History	X							
Concomitant disease	X							
Laboratory tests and auxiliary exams[3]	X		X	X	X	X	X	X
QoL score	X		X	X	X	X	X	X
Randomization		X						
Treatment		X	X		X	X		
Concomitant medication	X	X	X	X	X	X	X	X
Concomitant Therapy[4]	X	X	X	X	X	X	X	X
Adverse events	X	X	X	X	X	X	X	X

Basic information[1]: Demographic data of subjects (name, age, gender, race, etc.).

Vital Signs[2]: pulse rate, blood pressure, body temperature.

Laboratory tests and auxiliary exams[3]: Stage 0: complete blood count, urinalysis, urinary system ultrasound, bladder capacity, postvoid residual urine volume, and renal function, Serum hCG for women of childbearing age including within 1 year after menopause. Weeks 7 and 14: complete blood count, urinalysis, postvoid residual urine volume. Week 1,3,8, and 10: urinalysis.

Concomitant Therapy[4]: any treatment for OAB other than OAB medications.
