# Supplementary material for: A Multi-Center, Randomized, Blind, Controlled Clinical Trial of the Safety and Efficacy of Micro Radio Frequency Therapy System for the Treatment of Overactive Bladder
Source: Front Med (Lausanne). 2022 May 12;9:746064. doi: 10.3389/fmed.2022.746064 (PMC9133845; doi:10.3389/fmed.2022.746064)
Supplement: Supplementary file 4 [file Table_4.pdf]

Supplementary Table 4: discontinuation criteria

Investigator's decision to withdraw
Not meeting the inclusion criteria after enrollment.<br>Subject experiencing a serious adverse event.<br>Finding device too ineffective, not clinically significant.<br>The subject's condition, necessitating urgent medical, hospitalization, or surgical intervention.<br>The subject develops certain comorbidities, complications, or special physiological changes that make it inappropriate to continue the study.<br>Other conditions that the investigator considers inappropriate to continue to accept the clinical study.
Subject 's decision to withdraw
Subject has the right to withdraw from the study halfway as provided in the informed consent form.<br>（If the subject does not officially withdraw, but misses any follow-ups due to no further use of medical devices, relevant assessments and examinations, this case is also deemed as withdrawal.）
