# Supplementary material for: A Multi-Center, Randomized, Blind, Controlled Clinical Trial of the Safety and Efficacy of Micro Radio Frequency Therapy System for the Treatment of Overactive Bladder
Source: Front Med (Lausanne). 2022 May 12;9:746064. doi: 10.3389/fmed.2022.746064 (PMC9133845; doi:10.3389/fmed.2022.746064)
Supplement: Supplementary file 5 [file Table_5.pdf]

Supplementary Table 5: Baseline characteristics of the patients

Characteristics	Experimental group (n=76)	Control group (n=38)	T/χ2/Z	P
Age (year)	44.17±12.79	41.74±12.13	0.97	0.3319
Gender,F/M	63/13	34/4	0.8624	0.3526
Height (m)	1.60±0.06	1.61±0.07	0.16	0.8724
Weight (kg)	57.82±9.27	55.49±7.87	1.33	0.1863
Systolic blood pressure	123.03±14.66	118.58±13.76	1.56	0.1220
Diastolic blood pressure	76.26±10.75	73.76±10.39	1.18	0.2391
Daily voiding times	14.54±5.99	15.08±7.85	0.1594	0.8734
Daily UI episodes	0.44±1.83	0.44±1.83	0.2813	0.7785
Daily urgency episodes	12.85±6.89	12.85±6.89	0.7785	0.9067
Nightly nocturia episodes	2.60±1.62	2.58±2.22	0.6383	0.5233
Duration of disease△	3.24±2.56	2.83±2.88	1.3968	0.1625
Residual urine volume	19.86±23.59	14.76±14.44	0.4358	0.6630
Bladder capacity	241.32±112.19	231.74±106.03	0.1069	0,9149
B-scan ultrasonography				
Normal	54	25	0.3298	0.5658
Abnormal	22	13		
Gravidity				
Positive	0	0		
Normal	53	28		
Unchecked	10	6	0.915▲	0.633
Inapplicable to males patients	13	4		

The presented data as means ± standard deviations.

△The duration of disease was calculated in years.

▲The statistical calculation excludes unchecked and inapplicable to male patients.
