# Supplementary material for: A Multi-Center, Randomized, Blind, Controlled Clinical Trial of the Safety and Efficacy of Micro Radio Frequency Therapy System for the Treatment of Overactive Bladder
Source: Front Med (Lausanne). 2022 May 12;9:746064. doi: 10.3389/fmed.2022.746064 (PMC9133845; doi:10.3389/fmed.2022.746064)
Supplement: Supplementary file 6 [file Table_6.pdf]

Supplementary Table 6 Comparisons of the primary efficacy end points between the two groups

Dataset	Variable	Experimental group (n=76)	Control group (n=38)	Total (N=114)	χ2	P
FAS	effective rate					
	Invalid	25(32.9%)	28(73.7%)	53(46.5%)	16.943	0.000
	valid	51(67.1%)	10(26.3%)	61(53.5%)		
PPS	effective rate					
	Invalid	16(23.9%)	28(73.7%)	44(41.9%)	24.705	0.000
	valid	51(76.1%)	10(26.3%)	61(58.1%)		
SA	effective rate					
	Invalid	30(39.5%)	28(73.7%)	58(50.9%)	11.863	0.001
	valid	46(60.5%)	10(26.3%)	56(49.1%)		
