# Supplementary material for: A Multi-Center, Randomized, Blind, Controlled Clinical Trial of the Safety and Efficacy of Micro Radio Frequency Therapy System for the Treatment of Overactive Bladder
Source: Front Med (Lausanne). 2022 May 12;9:746064. doi: 10.3389/fmed.2022.746064 (PMC9133845; doi:10.3389/fmed.2022.746064)
Supplement: Supplementary file 7 [file Table_7.pdf]

Supplementary Table 7 Comparisons of the Secondary Efficacy End Points between the two groups（FAS）

Variable		Experimental group	Control group	Z	P
Daily voids	N(NMISS）	69 (7)	38 (0)		
	Mean±SD	10.38±4.29	13.51±6.87	2.7164	0.0066
Daily UI episodes	N(NMISS）	69 (7)	38 (0)		
	Mean±SD	0.28±0.99	0.48±1.51	0.4991	0.6177
Daily urgency episodes	N(NMISS）	69 (7)	38 (0)		
	Mean±SD	7.44±4.64	12.26±7.64	3.5438	0.0004
Nightly nocturia episodes	N(NMISS）	69 (7)	38 (0)		
	Mean±SD	1.44±1.12	2.07±1.72	2.1054	0.0353
Residual urine volume	N(NMISS）	68(8)	38(0)		
	Mean±SD	8.02±13.85	8.87±16.56	0.2440	0.8072
Quality of life score	N(NMISS）	69 (7)	38 (0)		
	Mean±SD	-2.11±1.49	-0.60±0.86	5.3453	<0.0001
