# Supplementary material for: A Multi-Center, Randomized, Blind, Controlled Clinical Trial of the Safety and Efficacy of Micro Radio Frequency Therapy System for the Treatment of Overactive Bladder
Source: Front Med (Lausanne). 2022 May 12;9:746064. doi: 10.3389/fmed.2022.746064 (PMC9133845; doi:10.3389/fmed.2022.746064)
Supplement: Supplementary file 8 [file Table_8.pdf]

Supplementary Table 8 Proportion of patients reporting adverse events (AE) through 14 weeks with radiofrequency therapy system.

Variable	Experimental group (n=76)		Control group (n=38)		Total (N=114)		p-value
Any serious AE	0 (0.0)	0	1 (2.6)	1	1 (0.9)	1	0.333^
Any device- or procedure-related AE	9 (11.8)	9	10 (26.3)	10	19 (16.7)	19	0.064^
Any AE	22 (28.9)	24	19 (50.0)	23	41 (36.0)	47	0.038^
Urinary tract infection	12 (15.8)	13	12 (31.6)	14	24 (21.1)	27	0.086^
Urine leukocytosis	1 (1.3)	1	2 (5.3)	2	3 (2.6)	3	0.257^
Urethrorrhagia	2 (2.6)	2	0 (0.0)	0	2 (1.8)	2	0.552^
Urethral injury	1 (1.3)	1	1 (2.6)	1	2 (1.8)	2	>0.999^
Hematuria	1 (1.3)	1	1 (2.6)	1	2 (1.8)	2	>0.999^
（left）Tinnitus	0 (0.0)	0	1 (2.6)	1	1 (0.9)	1	0.333^
Anaphylactic rhinitis	0 (0.0)	0	1 (2.6)	1	1 (0.9)	1	0.333^
Mixed hemorrhoid	0 (0.0)	0	1 (2.6)	1	1 (0.9)	1	0.333^
Cervicodynia	1 (1.3)	1	0 (0.0)	0	1 (0.9)	1	>0.999^
Cervical disc herniation	1 (1.3)	1	0 (0.0)	0	1 (0.9)	1	>0.999^
Chronic pharyngitis	1 (1.3)	1	0 (0.0)	0	1 (0.9)	1	>0.999^
Urethrostenosis	1 (1.3)	1	0 (0.0)	0	1 (0.9)	1	>0.999^
Uroschesis	1 (1.3)	1	0 (0.0)	0	1 (0.9)	1	>0.999^
Skin mass	0 (0.0)	0	1 (2.6)	1	1 (0.9)	1	0.333^
Diagnosis of breast cancer	1 (1.3)	1	0 (0.0)	0	1 (0.9)	1	>0.999^
Insomnia	0 (0.0)	0	1 (2.6)	1	1 (0.9)	1	0.333^

AE, adverse event.

Values were presented as number of cases(incidence)cases.

^Fisher test
